# QuEChERS-Based Approach to the Extraction of Five Calcium Channel Blockers from Plasma Determined by UPLC-MS/MS

**DOI:** 10.3390/molecules28020671

**Published:** 2023-01-09

**Authors:** Tingting Zhao, Wen Jiang, Xiaolan Zhen, Chengcheng Jin, Yifan Zhang, Hui Li

**Affiliations:** 1 College of Pharmacy, Hebei Medical University, Shijiazhuang 050000, China; 2 College of Chemistry and Pharmaceutical Engineering, Hebei University of Science and Technology, Shijiazhuang 050000, China; 3 Hebei Institute of Drug and Medical Device Inspection, Shijiazhuang 050000, China

**Keywords:** calcium channel blockers, QuEChERS, UPLC-MS/MS, human plasma, hypertension

## Abstract

Here, a QuEChERS (quick, easy, cheap, effective, rugged, and safe) pretreatment method was combined with UPLC-MS/MS to facilitate the rapid and reliable simultaneous detection of five calcium channel blockers (CCBs) in human plasma. For this approach, samples were treated with 1 mL of acetonitrile, 350 mg of magnesium sulfate, and 70 mg of PSA adsorbent prior to centrifugation. Supernatants then underwent gradient elution for 8 min with an Agilent C18 column using an acetonitrile-water solution supplemented with 5 mmol⋅L^−1^ of ammonium acetate. This technique exhibited a good linear response in the 1–800 ng⋅mL^−1^ range for the analyzed drugs, with an R^2^≥ 0.9921, an accuracy of 87.54–113.05%, a matrix effect (ME) of 91.21–116.39%, a precision of 0.19–11.64%, and stability of no more than 10.05%. This time-saving QuEChERS reagent-based pretreatment technique thus allowed for the simultaneous and accurate detection of five CCBs in human plasma samples, providing a promising new basis for therapeutic drug monitoring in patients with hypertension.

## 1. Introduction

Hypertension is a condition in which patients experienced persistently elevated systemic blood pressure levels outside the normal physiological range, elevating the risk of a range of renal cardiac, cerebral, and peripheral vascular diseases. Achieving sustained blood pressure control in hypertensive patients often necessitates pharmacologic intervention using a range of first-line treatments including angiotensin-converting enzyme inhibitors, angiotensin receptor blockers, beta-blockers, calcium channel blockers (CCBs), and diuretics.

CCBs are prescribed primarily for the treatment of peripheral vasospasm, migraine headaches, angina pectoris, and arterial hypertension [1]. CCB use rates have risen in recent decades such that they now account for over 30% of hypotensive drug use in patients affected by a range of cardiovascular conditions [2]. However, even when care is taken, these calcium antagonists can cause toxic effects resulting in sudden illness or death [3]. Some reports suggest that CCB use is among the most significant factors contributing to therapeutic cardiovascular drug poisoning and mortality [4].

Based on their affinity and effects on the heart and arterial vessels, these drugs are classified as dihydropyridine CCBs, which primarily affect the vascular smooth muscle, and non-dihydropyridine CCBs, which exhibit specific targeting of myocardial L-type channels [5]. At therapeutic doses, dihydropyridines can thus promote vasodilation [6,7]. Pharmacological selectivity may be lost when toxic levels of these drugs are consumed [8], resulting in potentially severe adverse reactions including low blood pressure, nausea, vomiting, rhythm depression, and cardiac arrest in severe cases [9]. Maintaining an appropriate plasma concentration of these drugs is thus vital to ensure that optimal patient blood levels are maintained and that a proper dose is utilized.

Liquid chromatography-mass spectrometry (LC-MS) approaches are among the most widely applied strategies for the detection of a diverse range of compounds, and they are the primary strategy employed to analyze calcium antagonists in bio-samples including serum, plasma, and milk owing to their excellent sensitivity and acquisition speed [10,11,12,13,14]. The combination of high-performance liquid chromatography and ultraviolet detection (HPLC-UV) [11], gas chromatography-tandem mass spectrometry (GC-MS) [15], and GC with electron-capture detection (GC-ECD) have also been used in various research settings. However, LC-based strategies alone cannot provide sufficient sensitivity when detecting many compounds, given that samples containing complex substrates inevitably contain many spurious peaks for which effective separation cannot be achieved. The GC-MS-based detection of pharmaceutical compounds also necessitates derivatization and time-consuming sample processing.

Appropriate analytical sample preparation is essential to mitigate interference caused by endogenous compounds or drug metabolites when biological samples consist of a complex matrix likely to disturb analyses. To date, many extraction techniques have been designed including protein precipitation (PP) [12], liquid-liquid extraction (LLE) [14], solid-phase extraction (SPE) [11,13], solid-phase microextraction (SPME), and others have been developed and used to measure drug concentrations in blood samples. However, these techniques are subject to certain limitations including high costs and complex workflow techniques. LLE can result in emulsification and consequent drug loss, while SPE requires expensive equipment and complex pre-column derivatization procedures that can impact the subsequent accuracy of quantitative analyses. In this study, a modified QuEChERS approach was used for sample fabrication. This methodological approach was initially published by Anastassiades et al. [16] and was based on SPE and matrix solid-phase dispersion (MSPD) techniques [17,18,19]. This improved strategy consists of three primary steps: (1) Homogenous sample extraction using organic solvents, (2) The separation of the organic layer by adding extracted samples to appropriate inorganic salts, and (3) The purification of specific analytes with an adsorbent [20]. Relative to traditional sample extraction techniques, optimized QuEChERS methods are more efficient, easier to complete, and less expensive.

The present study was developed with the goal of combining the performance of LC-MS/MS detection strategies and the utility of QuEChERS techniques in an effort to enable the rapid and simultaneous quantitative detection of amlodipine, nifedipine, nimodipine, nifedipine, and felodipine in human plasma samples. The resultant protocol only requires tiny samples and easy-to-implement pretreatment procedures, offering a reliable approach to therapeutic drug monitoring in individuals using calcium antagonists.

## 2. Results

### 2.1. Methodological Validation

#### 2.1.1. Selectivity

**Figure 1:**
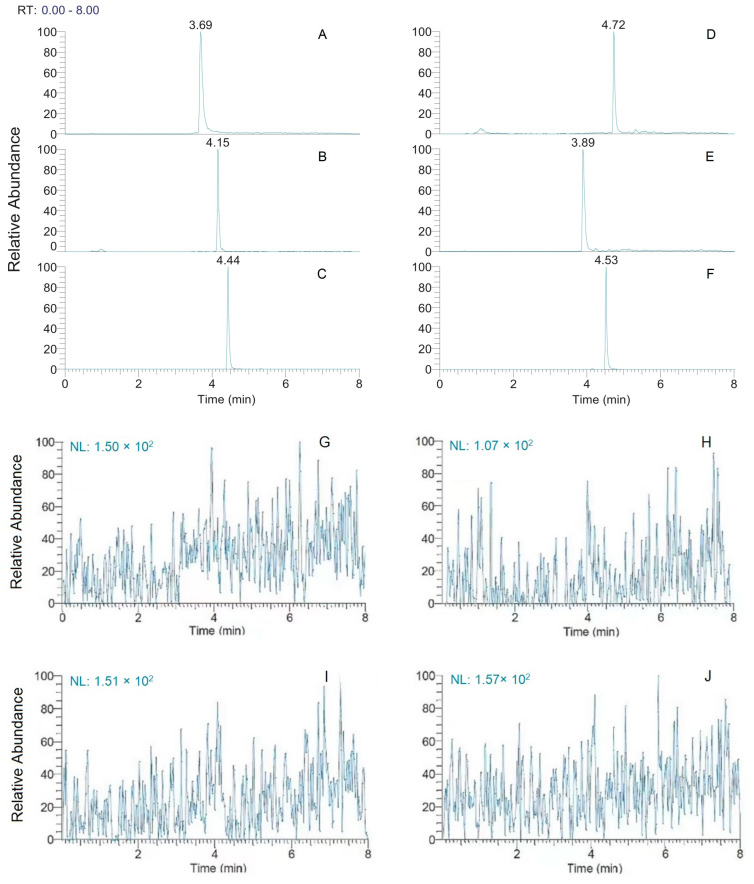

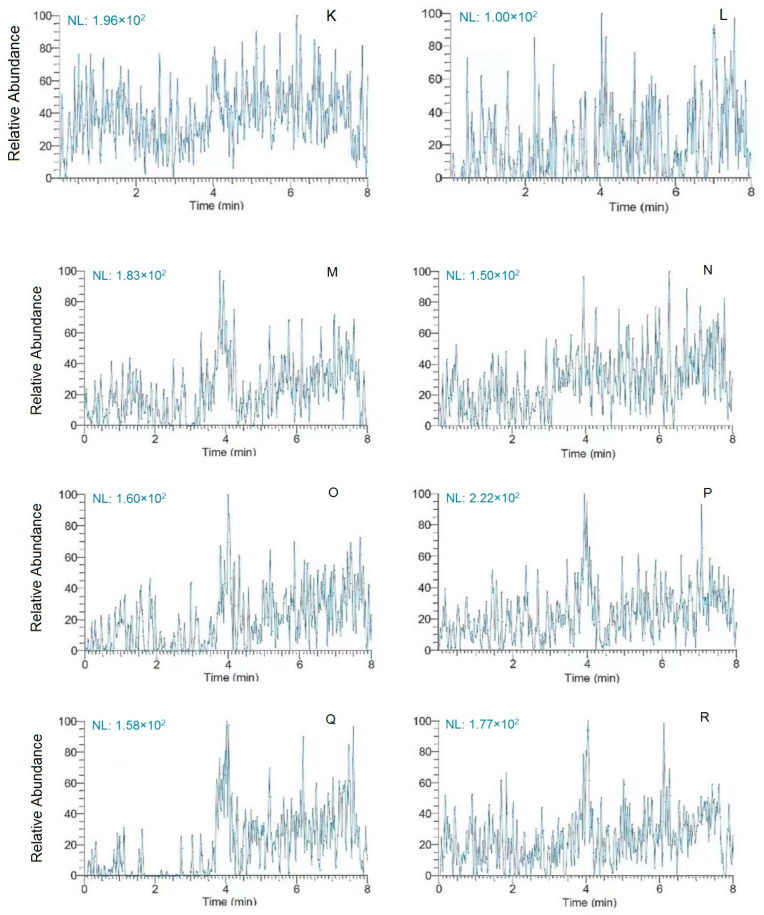
Typical chromatograms of IS-propranolol (**A**), nifedipine (**B**), nitrendipine (**C**), felodipine (**D**), amlodipine (**E**), and nimodipine (**F**) in blank plasma samples, the chromatogram of sixblank plasma samples (**G–N**) and chromatogram of six blank samples after HQC sample analysis (**M**–**R**).

No interference was detected when assessing blank plasma samples. Chromatograms for samples spiked with internal standard (IS), when extracted separately from CCBs and IS, did not exhibit any interfering retention time peaks and presented with good quantitative peak shape. IS concentrations were as follows:20 ng⋅mL^−1^ for amlodipine (AML) and nimodipine (NIM); 10 ng⋅mL^−1^ for nifedipine (NIF); 60 ng⋅mL^−1^ for nitrendipine (NIT); 200 ng⋅mL^−1^ for felodipine (FEL); 10 ng⋅mL^−1^ for IS (Figure 1). Carryover in the chromatogram of the blank plasma sample after high-concentration samples was barely visible, ensuring that the accuracy and precision of the method were not compromised. Chromatograms of blank plasma samples evaluating selectivity and carryover are shown in Figure 1G–R.

#### 2.1.2. Linearity

Peak area ratios for these five CCBs were linear within the 2.0–800 ng⋅mL^−1^ range, with each exhibiting a linear correlation coefficient (R^2^) > 0.992. Peak concentrations up to three times the baseline noise level were defined as LODs, while peak concentrations up to 10 times the baseline noise level were defined as LOQs. For details regarding these specific parameters, see Table 1.

#### 2.1.3. Accuracy and Precision

The accuracy of this method ranged from 87.54–113.05%, with inter-day precision ranging from 0.19–11.04% and intra-day precision ranging from 0.28–11.64%. For further detail regarding these parameters, see Table 2.

#### 2.1.4. Matrix Effect

The internal standard normalized MF for these five CCBs ranged from 91.21–116.39%, suggesting that these matrix effects are not likely to impact the determinations made using this method. It is generally stipulated that the extraction recovery rate should be greater than 50%, and the extraction recovery rate of this method is between 91.74–106.17%, which meets the requirements. For further details regarding specific values, see Table 2.

#### 2.1.5. Stability

The RSD value corresponding to short-term stability was less than or equal to 10.05%, and sample tray stability was less than or equal to 7.00%. Long-term storage stability was less than or equal to 9.41%. For further details see Table 3.

## 3. Discussion

### 3.1. Extraction Procedure Optimization

**Figure 2:**
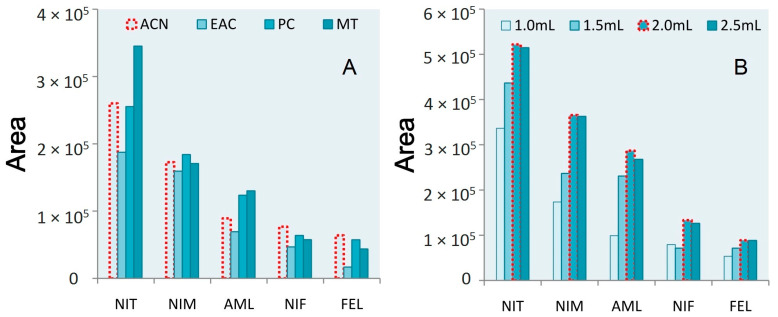
(**A**) The area results of five CCBs treated with different extraction solvents. (**B**) The area results of five CCBs treated with different volumes of extractants.

While structurally related, these different dihydropyridine drugs are all distinct from one another such that their relative solubility differs in particular solvents. The extraction effects for methanol (MT), ethyl acetate (EAC), acetonitrile (ACN), and acetone (CP) were therefore explored based on the area index values. Of these four tested extraction reagents, ethyl acetate yielded the smallest area value for each of these CCBs, while methanol exhibited the best extraction effects for nitrendipine, nimodipine, and amlodipine, although its efficacy was relatively poor for the other two drugs. Acetone and acetonitrile exhibited good performance for most of these CCBs, but slightly poorer for amlodipine. Of these two extraction reagents, the area values for drugs purified using acetone were lower than when using acetonitrile. Accordingly, subsequent experiments were performed using acetonitrile (Figure 2A).

Different acetonitrile volumes were next combined with samples, given the optimized extraction reagent usage. As shown in Figure 2B, the use of 2 mL of acetonitrile yielded larger peak area values.

### 3.2. Purification Reagent Selection

**Figure 3:**
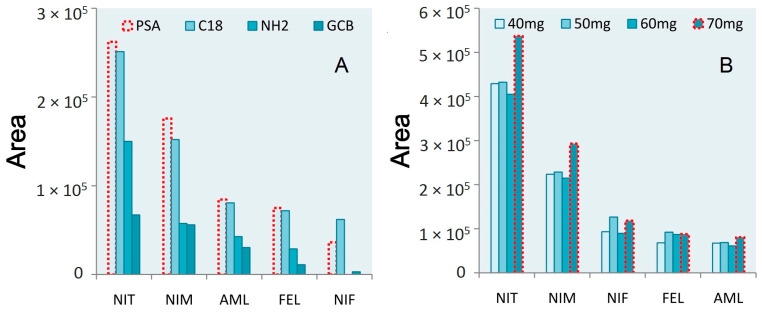
(**A**) The area results of five CCBs treated with different purifying agents. (**B**) The area results of five CCBs treated with different amounts of purifying agents.

The four purifying agents include g primary–secondary amine (PSA), C18, graphitized carbon black (GCB), and NH_2_. Area values were used to assess the relative performance of these different agents. PSA exhibited excellent purification efficacy for most tested CCBs in the sample matrix, although C18 exhibited better performance for nifedipine. However, peak areas for the four other analyzed CCBs were lower when using C18 than PSA. The overall rank order purification efficacy was as follows: PSA > C18 > NH_2_ > GCB (Figure 3A).

To better optimize absorbant dosing, four different amounts of PSA (40, 50, 60, and 70 mg) were tested. For these five CCBs, the most prominent peak areas were observed for nitrendipine, nimodipine, and amlodipine when using 70 mg of PSA. In contrast, better responses were observed for the other drugs when using 50 mg of PSA. When pretreatment was performed using 70 mg of PSA, nifedipine and felodipine exhibited smaller peak area values than when 50 mg of PSA was added. As such, 70 mg of PSA was used as an adsorbent in subsequent analyses of these five CCBs (Figure 3B).

### 3.3. Salting Reagent Selection

**Figure 4:**
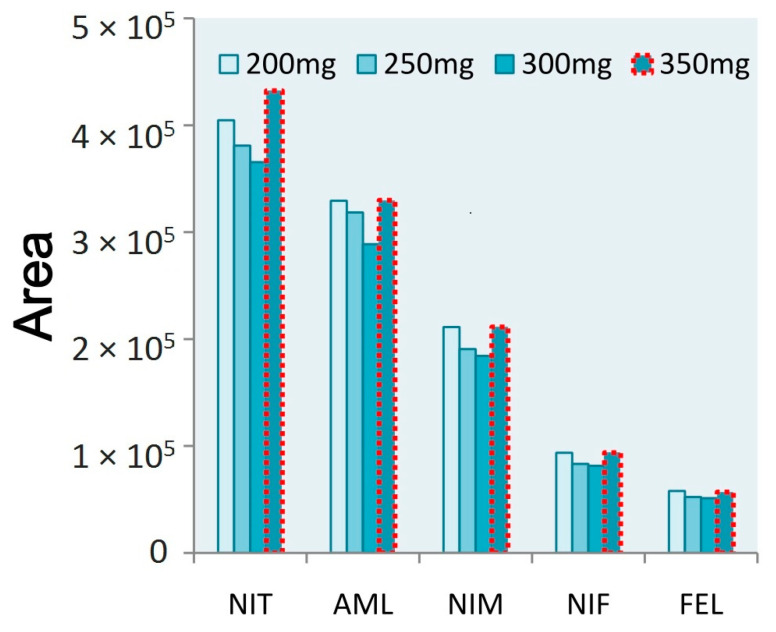
The area results of five CCBs treated with different amounts of MgSO_4_.

Based on the results of this study, anhydrous MgSO_4_ was selected as the salting agent. Different concentrations were selected for extraction (250, 300, 350, and 400 mg), with optimal results established based on area values. The highest CCB peak responses were evident when using 350 mg of anhydrous magnesium sulfate. A slightly less pronounced peak response was observed at a 200 mg dose. As the extraction dose was the most critical factor, a salt dose of 350 mg was selected (Figure 4).

### 3.4. Comparisons

Most prior studies have primarily focused on a single calcium antagonist, whereas few have described the simultaneous detection of multiple CCBs. In this study, five calcium antagonists were determined simultaneously by QuEChERS-UPLC-MS/MS method for the first time. While these dipine-drugs are derived from the same family, the structural differences in their specific moieties give them each unique physicochemical characteristics. The methodological approach to their detection is also subject to bio-matrix complexity and the low expected concentrations of these compounds in some instances. Our work is challenging.

Few methods for the simultaneous determination of five calcium antagonists have been established in the last five years of research. Most of the methods in Table 4 measured one-three drugs and included only one calcium antagonist. The method we established allows simultaneous determination of five CCBs, which indicates that our method covers an extensive range of drugs and is more applicable. The developed method has LOD and LOQ of 0.014, 0.048 for AML, and 0.007,0.025 for NIM. Existing studies have shown LOD and LOQ greater than 0.2 and 0.5 for AML [11,12] and greater than 0.05 and 0.12 for NIM [13,14]. The new method demonstrated lower LOD and LOQ and, therefore, has higher sensitivity than published methods. The extraction recoveries of each drug in this method were more than 94%, which was higher than those of the corresponding drugs in other studies in Table 4.

Overall, this improved QuEChERS-UPLC-MS/MS method achieved good recovery, precision, and accuracy while maintaining good efficacy and cost-effectiveness, thereby supporting greater environmental conservation. For further comparisons of these different parameters, see Table 4.

In previous work conducted by our group, we applied the QuEChERS pre-treatment technique to assess the concentrations of multiple drug classes. Analyses of multiple tyrosine kinase inhibitors (TKI) concentrations in human plasma [22] and multiple immunosuppressant concentrations in whole human blood [23] have performed well in terms of LOQ and recovery as compared to other assays for similar drugs. In addition, other members ofour group have attempted to use the QuEChERS technique in combination with LC-MS to detect 16illicitly added drugs in capsule dietary supplements containing lipid-lowering drugs, diuretics, and appetite suppressants [24]. They successfully verified the utility of this method as a means of detecting and quantifying the target analytes in dietary supplement samples. Therefore, we propose that this QuEChERS technique applies not only to the field of calcium antagonists but also to a broad rangeof therapeutic drug monitoring applications.

## 4. Materials and Methods

### 4.1. Chemicals and Reagents

All CCBs and propranolol hydrochloride were acquired from National Institutes for Food and Drug Control (Beijing, China) and were ≥99.2% pure. Methanol (MT), ethyl acetate (EAC), acetonitrile (ACN), and acetone (CP) were obtained from Fisher Chemical (Shanghai, China). Ammonium acetate was obtained from the Tianjin BODI Chemicals Co., Ltd. (Tianjin, China). Analytical grade magnesium sulfate anhydrous (MgSO_4_) was from Kemiou Chemical Reagent Co., Ltd. (Tianjin, China). QuEChERS adsorbents were provided by Agilent(Tianjin, China). Ultrapure water was from Watsons.

### 4.2. Instrument and Analytical Conditions

**Figure 5:**
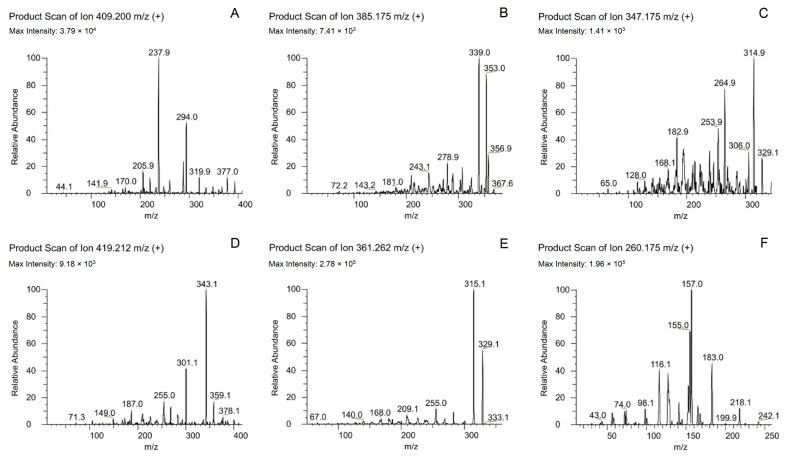
Product ion mass spectra of amlodipine (**A**), felodipine (**B**), nifedipine (**C**), nimodipine (**D**), nitrendipine (**E**) and IS-propranolol (**F**) in positive mode.

**Figure 6:**
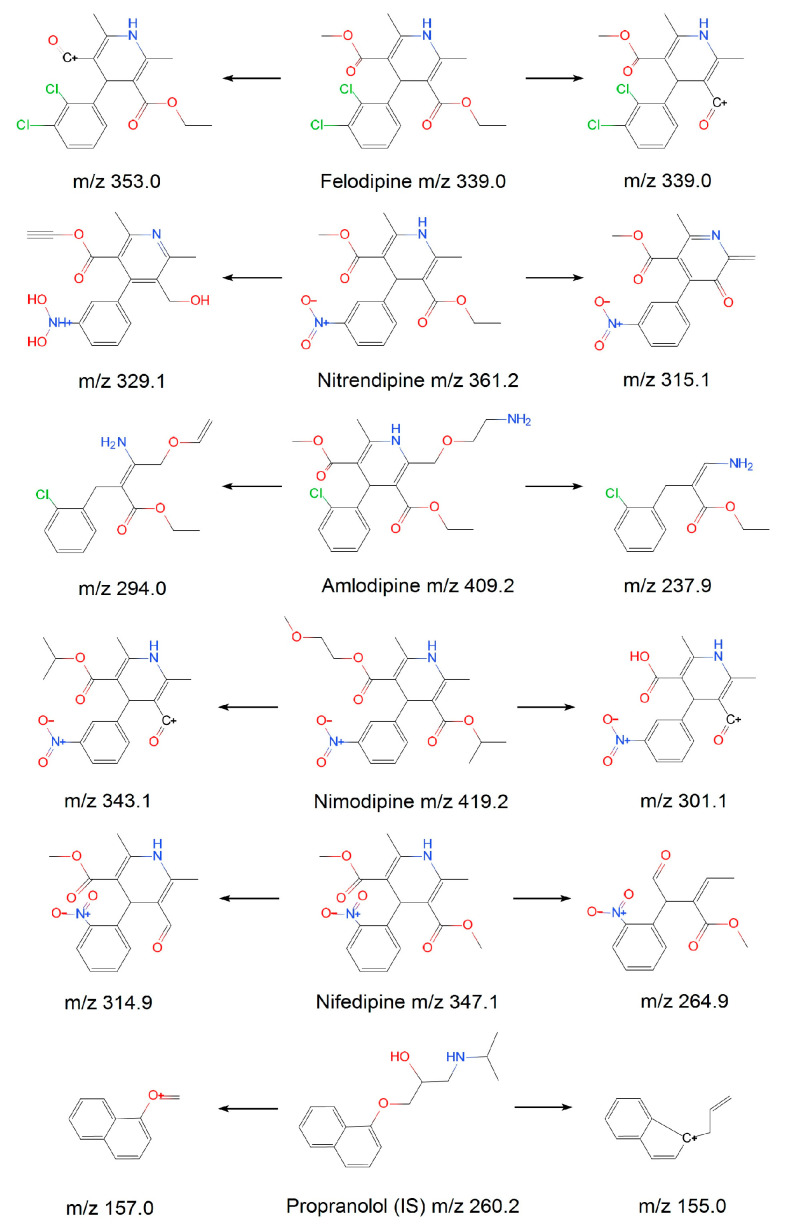
The chemical structure and possible fragmentation pathways of five CCBs.

A VanquishTM Flex ultra-HPLC System coupled with a TSQ Altis^TM^ Triple Quadrupole MS Instrument (Thermo Fisher Scientific, Waltham, Massachusetts USA) was used for all analyses. Briefly, samples (5 μL) were spiked into a Poroshell 120 EC-C18 (column temperature 45 °C). Elution was performed using 5 mmol/L ammonium acetate–water (A) and acetonitrile (B) at a 0.3 mL⋅min^−1^ flow rate using the following settings: 0–1.0 min 20% B; 1.0–3.0 min, linear gradient from 20–90% B; 3.0–6.0 min, 90% B; 6.0–6.1 min, 90–20% B; 6.1–8.0 min, 20% B. The relevant MS parameters were positive mode electrospray ionization source (ESI+); selection reaction monitoring (SRM). Mass transitions of the RF Lens, CE, and RT, are presented in Table 5. Product spectra for each analyte are presented in Figure 5. The chemical structure and possible fragmentation pathways of each compound are shown in Figure 6.

### 4.3. Solution Preparation

Each drug and the internal standard (IS) were prepared as a 100 μg⋅mL^−1^stock solution in methanol. Methanol was then used to dilute these drugs to appropriate working concentrations including NIF:1, 2.5, 5, 10, 25, 40 ng⋅mL^−1^; AML and NIM: 2, 5, 10, 20, 50, 80 ng⋅mL^−1^; NIT: 6, 15, 30, 60, 150, 240 ng⋅mL^−1^; FEL: 20, 50, 100, 200, 500, 800 ng⋅mL^−1^; Propranolol (IS):10 ng⋅mL^−1^.

### 4.4. Plasma Sample Preparation

Plasma samples were collected from healthy human donors and prepared via centrifugation prior to storage at −110 °C. To prepare samples, 50 μL of human plasma was added to a 2 mL polypropylene tube, after which 100 μL each of the IS methanol solution and the mixed standard preparation were added to the same tube and mixed thoroughly. Then, 1 mL of acetonitrile, 350 mg of MgSO4, and 70 mg of PSA were added to the tube in sequence followed by vigorous shaking for 1 min. The entire mixture was taken by centrifugation for 5 min at 1500× *g* at 4 °C, after which the residue was re-dissolved in 500μLof methanol after being dried under a nitrogen stream. The solution was then passed through a filter membrane using a syringe. The Ethics Committee of Hebei Medical University approved this study.

### 4.5. Methodological Validation

The readouts assessed to ensure the suitability and efficacy of the developed methods included selectivity, calibration curves, accuracy, precision, analyte stability in biological matrices, and matrix effects, which were analyzed as detailed below.

#### 4.5.1. Selectivity and Carryover

Six blank plasma samples of healthy people and sixplasma samples containing internal standard and standard solution were analyzed to assess methodological selectivity. Responses of interfering components below 20% of the LLOQ for the analyte and 5% of the internal standard are acceptable.

Validation of carryover was assessed by injecting a blank sample after the high-concentration sample. The carryover of the high-concentration standard sample in the blank sample should not exceed 20% of the LLOQ and 5% of the internal standard.

#### 4.5.2. Linearity

Following pretreatment using the developed analytical protocol, a series of calibration samples were analyzed. Linear regression analyses were performed with the Xcalibur system based on the ratio of the detection response for the five CCBs to the response of the IS (dependent variable, y) graphed against the drug concentration in prepared calibration samples (independent variable, x).

#### 4.5.3. Precision and Accuracy

To assess accuracy, QC samples prepared at low, medium, and high concentrations were analyzed, with these analyses being repeated three times for each sample concentration. Intra-day precision was measured by replicating this analysis three times per day at all sample concentrations, while inter-day precision was calculated by repeating three analyses of QC samples on six successive days.

#### 4.5.4. Stability

Stability was validated using QC samples prepared at different concentrations. Short-term stability was assessed after storage for 24 h at room temperature (25 °C), while long-term stability was assessed following storage in the sample tray (4 °C) for 24 or 48 h, or after storage in refrigerator-frozen storage (−20 °C) for 7 days.

#### 4.5.5. Matrix Effect and Extraction Recovery

QC samples, standard analyte solutions, and IS standard solutions were evaluated to assess the matrix effect (ME). The matrix factor (MF) was determined based on the sample peak area’s ratio to the standard solution’s peak area. The normalized matrix factor (MF_i_) was determined by dividing the MF for a given analyte by the MF for the IS.

The extraction recovery (ER) was calculated by the ratio of the area of the QC sample to the area of the blank sample added with the corresponding concentration standard solution after pretreatment.

## 5. Conclusions

Here, an LC-MS/MS strategy was successfully combined with an optimized QuEChERS pretreatment technique in order to simultaneously measure levels of amlodipine, nimodipine, nifedipine, nifedipine, and felodipine in samples of human plasma. Plasma pretreatment was straightforward, and the use of pre-weighed QuEChERS reagents can reduce labor and time requirements while also being amenable to use as a pretreatment for analyses of other compounds of interest. This approach achieved an accuracy of 87.54–113.05%, precision from 0.19–11.64%, extraction recovery of 94.85–97.32%,and a stability RSD value ≤ 10.05%, with negligible matrix interference. This method is thus an effective and straightforward approach to therapeutic drug monitoring focused on calcium antagonists.

## Figures and Tables

**Table 1:** Regression equations, LODs, and LOQs of compounds.

Compounds	Linear Equation	Linear Range (ng·mL^−1^)	R^2^	LODs (ng·mL^−1^)	LOQs (ng·mL^−1^)
AML	*Y* = −0.171406 + 0.0869037*x*	2.0–80	0.9951	0.014	0.048
FEL	*Y* = −0.0524462 + 0.00288808*x*	20–800	0.9933	0.220	0.732
NIF	*Y* = −0.429218 + 0.421058*x*	1.0–40	0.9921	0.002	0.006
NIM	*Y* = −0.107548 + 0.0537931*x*	2.0–80	0.9914	0.007	0.025
NIR	*Y* = −0.0401095 + 0.0169601*x*	6.0–240	0.9947	0.041	0.137

**Table 2:** ME, extraction recovery, accuracy, and precision of five compounds (*n* = 3).

Compounds	Spiked (ng·mL^−1^)	ME (%)	ER (%)	Accuracy (%)	Precision (RSD %)	
					Inter-Day	Intra-Day
AML	5	102.06	95.23	101.43	0.69	2.00
	20	99.64	102.67	105.83	6.89	1.81
	80	93.59	94.06	87.54	2.24	2.86
FEL	50	97.18	106.17	103.95	3.35	0.64
	200	94.52	94.86	106.15	11.04	2.87
	800	91.21	96.41	90.15	6.36	2.61
NIF	2.5	91.23	100.80	111.23	0.82	0.28
	10	98.19	95.98	96.27	3.79	0.71
	40	92.43	91.74	91.60	4.45	2.10
NIM	5	93.66	91.94	104.18	0.21	1.45
	20	110.48	95.18	103.35	8.51	1.85
	80	95.09	97.45	92.91	5.71	11.64
NIT	15	99.11	97.55	113.05	4.43	2.57
	60	116.39	98.45	105.08	0.19	1.66
	240	108.84	94.78	96.63	3.22	2.75

**Table 3:** The stability of the QC samples under four different conditions (*n* = 3).

Compounds	Spiked (ng·mL^−1^)	Stability (RSD%)			
		25 °C/24 h	4 °C/24 h	4 °C/48 h	−20 °C/7d
AML	5	1.52	1.44	2.69	6.33
	20	2.30	1.61	0.95	0.69
	80	1.50	0.48	1.62	3.36
FEL	50	6.11	2.13	4.77	5.38
	200	1.96	3.19	7.00	4.72
	800	5.47	0.85	2.45	9.41
NIF	2.5	2.16	1.43	1.64	2.34
	10	7.42	0.57	0.92	1.23
	40	0.91	1.26	0.37	4.53
NIM	5	4.95	1.70	5.58	2.93
	20	3.02	1.20	2.40	4.50
	80	0.50	5.41	3.27	7.60
NIT	15	2.16	1.70	1.55	8.08
	60	10.05	1.06	6.42	2.02
	240	2.50	3.64	2.75	9.10

**Table 4:** Comparison of the proposed method with other published methods for the quantitative detection of CCBs.

Method	Analyte	LOD(ng/mL)	LOQ(ng/mL)	ER(%)	Ref.
QuEChERS/UPLC-MS/MS	AML	0.014	0.04	97.32	This work
	FEL	0.220	80.732	96.15	
	NIF	0.002	0.006	96.17	
	NIM	0.007	0.025	94.85	
	NIT	0.041	0.137	96.93	
PP/first derivative SFS	AML	1.160	3.516	95.20	[21]
HPLC-MS/MS	AML	0.2	0.5	50.90	[11]
MSPE/HPLC-UV	NIM	0.28	0.84	59.87	[12]
PP/SFC-MS/MS	NIM	0.05	0.12	91	[13]
SPE/LC-MS/MS	FEL	-	0.59	91.9	[14]
LLE/LC-MS/MS	NIT	-	-	89.51	[15]

**Table 5:** The MS/MS fragment ions, fragmentor voltage, collision voltage, and retention time of the five calcium blockers.

Compounds	Precursor (*m*/*z*)	Product (*m*/*z*)	Collision Energy(V)	RFLens(V)	Retention Time (min)
AML	409.200	237.9	12.03	53	3.89
		294.0	12.20		
FEL	385.175	339.0	13.13	53	4.72
		353.0	13.55		
NIF	347.175	264.9	12.75	46	4.15
		314.9	8.79		
NIM	419.212	301.1	20.60	55	4.53
		343.1	10.64		
NIR	361.262	315.1	13.17	64	4.44
		329.1	13.72		
PRO	260.175	155.0	25.30	47	3.69
		157.0	20.12		

## Data Availability

Data is contained within the article.

## Sample Availability

Samples of the compounds (AML, NIF, NIT, NIM, FEL, Propranolol) are available from the authors.
